# Short Sleep Duration Is Associated with Risk of Future Diabetes but Not Cardiovascular Disease: a Prospective Study and Meta-Analysis

**DOI:** 10.1371/journal.pone.0082305

**Published:** 2013-11-25

**Authors:** Elizabeth G. Holliday, Christopher A. Magee, Leonard Kritharides, Emily Banks, John Attia

**Affiliations:** 1 Centre for Clinical Epidemiology and Biostatistics, School of Medicine and Public Health and Hunter Medical Research Institute, University of Newcastle, Newcastle, New South Wales, Australia; 2 Centre for Health Initiatives, University of Wollongong, Wollongong, New South Wales, Australia; 3 Department of Cardiology, Concord Repatriation General Hospital, The University of Sydney, Sydney, New South Wales, Australia; 4 National Centre for Epidemiology and Population Health, Australian National University, Canberra, Australian Capital Territory, Australia; 5 The Sax Institute, Sydney, New South Wales, Australia; Hospital General Dr. Manuel Gea González, Mexico

## Abstract

Epidemiologic studies have observed association between short sleep duration and both cardiovascular disease (CVD) and type 2 diabetes, although these results may reflect confounding by pre-existing illness. This study aimed to determine whether short sleep duration predicts future CVD or type 2 diabetes after accounting for baseline health. Baseline data for 241,949 adults were collected through the 45 and Up Study, an Australian prospective cohort study, with health outcomes identified via electronic database linkage. Cox proportional hazards models were used to estimate hazard ratios (HR) and 95% confidence intervals. Compared to 7h sleep, <6h sleep was associated with incident CVD in participants reporting ill-health at baseline (HR=1·38 [95% CI: 1·12-1·70]), but not after excluding those with baseline illness and adjusting for baseline health status (1·03 [0·88-1·21]). In contrast, the risk of incident type 2 diabetes was significantly increased in those with <6h versus 7h sleep, even after excluding those with baseline illness and adjusting for baseline health (HR=1·29 [1·08-1·53], P=0.004). This suggests the association is valid and does not simply reflect confounding or reverse causation. Meta-analysis of ten prospective studies including 447,124 participants also confirmed an association between short sleep and incident diabetes (1·33 [1·20-1·48]). Obtaining less than 6 hours of sleep each night (compared to 7 hours) may increase type 2 diabetes risk by approximately 30%.

## Introduction

The World Health Organization (WHO) recently published a global strategy to “prevent and control” major noncommunicable diseases such as CVD and type 2 diabetes, with a key emphasis upon reducing exposure to established risk factors [1]. A potential risk factor for cardiometabolic disease is sleep duration. Various epidemiologic studies have observed associations of either deficient or excessive sleep duration with cardiometabolic disease, albeit with uncertain causality. Given the burgeoning global prevalence of cardiovascular disease and type 2 diabetes, and high levels of voluntary and involuntary sleep curtailment, a causal effect of sleep duration upon cardiometabolic disease risk has considerable implications for public health.

In relation to whether sleep duration predicts future, incident CVD, prospective studies have produced conflicting results. Different studies have alternatively reported prediction of future CVD by long sleep duration alone [2,3], short sleep duration alone [4–6], both short and long sleep duration (U-shaped relationship) [7–10] and neither short nor long sleep duration [11,12]. Several individual studies have reported substantial attenuation or abolition of apparent associations after statistical adjustment for covariates [5,11,13], suggesting that apparent associations may reflect residual confounding. In contrast, prospective studies [14–19] and a meta-analysis [20] have provided more consistent evidence for a relationship specifically between short sleep duration (<5-6 h) and type 2 diabetes development, although many studies have been insufficiently powered to thoroughly examine potential confounding, especially confounding by baseline illness. 

We report the largest prospective study to examine longitudinal associations between baseline sleep duration and future, incident CVD and type 2 diabetes. Specifically, we sought to determine the extent to which observed associations could be explained by confounding or effect modification by pre-existing illness or functional impairment. 

## Materials and Methods

The study sample comprised participants in the 45 and Up Study, a cohort study of 241,949 adults residing in the state of New South Wales (NSW), Australia. Participants were randomly selected from the Medicare Australia enrolment database, with ~18% of those contacted joining the study. The Medicare Australia database lists all Australian citizens. Although not necessarily representative of the entire population, the Study includes information about a large, heterogeneous group of adults and provides robust meaningful comparisons of outcomes based on internal comparisons within the cohort [21]. Consenting participants completed a comprehensive questionnaire and provided written, informed consent for linkage of baseline data to health databases. Questionnaires are available at http://www.45andup.org.au. 

### Ethics Statement

The 45 and Up Study received ethics approval from the University of NSW Human Research Ethics Committee. Ethics approval to use the data for this report was obtained from the NSW Population and Health Services Ethics Committee. 

### Exposure and covariates

The self-report questionnaire included the question “About how many hours in each 24 hour day do you usually spend sleeping?” Continuous responses were recoded into six ordinal categories, consistent with existing research: <6 h, 6 h (i.e. 6-<7 h), 7 h (7-<8 h), 8 h (8-<9 h), 9 h (9-<10 h) and ≥10 h. Serious illness was defined as self-reported, doctor-diagnosed current cancer (past month treatment for any cancer excluding non-melanoma skin cancer), or previously diagnosed cancer, heart disease, stroke or diabetes (based on self-report and current medications). These definitions were chosen to follow the Charlson comorbidity index [22] as closely as possible. Physical function was measured using the Medical Outcomes Study Physical Functioning (MOS-PF) scale, using coding as previously described [23]. Baseline health status was nominally defined as “healthy” for individuals reporting no serious illness at baseline and MOS-PF ≥75, and “less healthy” for individuals reporting serious illness at baseline or MOS-PF <75. Additional covariates were also coded for inclusion in statistical models. These included gender, age in 5-year groups, education (<11 years, school certificate, higher school certificate, some tertiary education, or University degree or higher), marital status (single, widowed, divorced/separated, or married/de facto), residential remoteness (major city, regional area, or remote area), alcohol consumption (0, 1-7, 8-14, or >15 drinks per week), smoking status (never, former, or current), health insurance status (private insurance/veterans card or none), annual household pre-tax income (<$AU 10,000, $10,000-$30,000, $30,000-$70,000, or “I would rather not answer the question”), body mass index (<18·5, 18·5-25, 25-30, or >30) and sufficient physical activity (≥30 min per day or <30 min per day).

### Outcomes

Baseline data were linked to hospital admission data from the NSW Admitted Patient Data Collection (APDC: to June 2010) and mortality data from the NSW Registry of Births, Deaths and Marriages (RBDM: to June 2010). The NSW APDC chronicles all hospitalisations in NSW, including dates of admission and discharge, the primary diagnostic reason for admission, and any additional diagnoses present. Diagnoses were described using the International Statistical Classification of Diseases and Related Health Problems, 10th Revision, Australian Modification (ICD10-AM) [24]. Incident cardiovascular disease (CVD) was defined as hospitalisation due to ischaemic heart disease (primary diagnosis of ICD10 code prefix I20 – I25), ischaemic stroke (primary ICD10 code prefix I63) or peripheral vascular disease (primary ICD10 code prefix I74), excluding day-only admissions. Type 2 diabetes was defined as overnight hospital admission for any reason, where a type 2 diabetes diagnosis was recorded in one of the first twenty ICD10 field codes. We note that this approach will underestimate the incidence of type 2 diabetes, since those developing type 2 diabetes but not admitted to hospital during follow-up will not be identified.

### Statistical analyses

Multivariate associations between sleep duration and each clinical outcome were assessed using Cox proportional hazards models, with time-to-event specified as the outcome. For type 2 diabetes, time from enrolment to the first hospitalisation recording a type 2 diabetes diagnosis was treated as the approximate time to type 2 diabetes onset. For each outcome, three multivariate models were fitted. For all models, individuals missing data for sleep duration, age, sex, physical functioning or prior serious illness were excluded. Individuals reporting the specified outcome at baseline (CVD or type 2 diabetes) were also excluded from analyses of the respective outcome, to focus estimation on incident disease. We defined prevalent CVD as self-reported heart disease, stroke, or treatment for heart disease, and prevalent type 2 diabetes as self-reported diabetes or anti-diabetic medication use. Model 1 included all eligible participants and was adjusted only for age and sex. Model 2 included the same participants as Model 1, but was adjusted for all defined covariates (see Methods) explaining a significant proportion of outcome variation in a partial likelihood ratio test (nominal *P*<0·1). Multiplicative interaction terms between sleep duration and: a) sex and; b) baseline health status were also assessed, to investigate potential effect modification. If either interaction test reached *P*<0·1 in a partial likelihood ratio test, effects were reported separately for both categories of the covariate. Model 3 included the same comprehensive set of covariates and interaction terms as Model 2 but additionally excluded all participants reporting serious illness at baseline (current/past cancer, diagnosed/treated heart disease, stroke, or diabetes), to reduce the potential for confounding or reverse causation by co-morbid baseline illness. Multivariate models were built using a backward, step-wise approach previously described [25] and fitted using Stata v11.1 software. Each model satisfied the proportional hazards assumption. A flow diagram detailing the exclusions, sample size and covariates used in each analysis model is provided in Figure S1.

### Meta-analysis of prospective studies assessing the relationship between short sleep and incident diabetes

Previous prospective studies assessing the relationship of short sleep with incident diabetes were identified from the literature, by searching the MEDLINE and EMBASE databases using the terms “sleep duration” and “diabetes” and (“prospective” or “cohort” or “longitudinal”), as previously described [20]. To be included in the meta-analysis, prospective studies were required to address incident diabetes in multivariate analyses of at least 500 individuals. If multiple, multivariate models were fitted, we recorded the effect estimate least likely to have been confounded by baseline illness or reverse causation, based on covariate adjustment or sensitivity analyses. Effect estimates reported as odds ratios, hazard ratios or risk ratios were treated as equivalent, since effect estimates and outcome prevalences were low [26]. Pooling was performed via fixed-effect Mantel-Haenszel meta-analysis using Stata v11.1. Between-study heterogeneity was investigated using Cochran’s Q statistic and the *I* ^2^ metric. Publication bias was assessed from the funnel plot, using Egger’s regression test for asymmetry [27]. 

## Results

The baseline dataset included 241,949 adults aged 45 years and older, of which 29,561 were excluded due to missing data for sleep duration, age, sex, physical functioning or serious illness. Table 1 summarises sociodemographic and health characteristics by sleep duration for the remaining 212,388 participants. The majority reported 7 h (24%) or 8 h (40.7%) daily sleep. Sleep duration showed significant association with all sociodemographic and health-related covariates in univariate analyses (*P*<0·001; see Table 1). 

**Table 1:** Sociodemographic and health-related factors by self-reported sleep duration in the 45 and Up Study (*N*=212,388).

	**<6 h**	**6 h**	**7 h**	**8 h**	**9 h**	**≥10 h**	***P***
	*N*=7,311	*N*=25,487	*N*=50,979	*N*=86,502	*N*=25,616	*N*=16,493	
Age (mean ± SD)	64.4 ± 11.9	61.6 ± 10.9	60.1 ± 10.1	61.7 ± 10.6	64.6 ± 11.2	68.6 ± 12.1	<0.001
Male gender (%)	41.8	46.3	48.1	46.2	47.7	53.8	<0.001
Tertiary education (%)	41.4	54.1	62.3	57.9	54.4	44.8	<0.001
Married (%)	61.3	70.3	77.1	78.0	77.9	69.0	<0.001
Urban residence (%)	47.6	49.7	49.7	43.5	39.0	39.7	<0.001
Private health insurance (%)	51.4	64.3	72.5	68.6	65.1	53.6	<0.001
Sufficient physical activity (%)	59.6	67.2	72.3	70.7	70.0	53.7	<0.001
Current smoker (%)	11.0	9.2	6.8	6.3	5.6	8.4	<0.001
Current drinker (>1 per day) (%)	23.6	27.9	32.5	33.3	35.3	31.1	<0.001
Obese (%)	25.3	23.1	19.3	19.8	19.7	25.1	<0.001
Existing illness/functional limitation (%)	55.5	38.9	29.6	34.2	43.0	65.3	<0.001

Analyses of CVD using Model 1 and 2 included 181,544 individuals without prevalent CVD, of whom 5,814 were hospitalised for CVD during follow-up (mean duration: 2·3 y). Model 1, which was adjusted only for age and sex, suggested association of short sleep (<6 h: HR=1·16 [95% CI: 1·01-1·34], *P*=0·041), with incident CVD. Model 2, which incorporated adjustment for a comprehensive set of covariates, showed significant interaction between sleep duration and baseline health status upon CVD risk (*P*_interaction_=0·01), indicating heterogeneity of the sleep duration effect between the two health groups. Sub-group analyses revealed a significant association of short sleep (<6 h) with CVD in less healthy individuals (HR=1·38 [95% CI: 1·12-1·70], *P*=0·002), but not in healthy individuals (HR=0·92 [0·75-1·14]). Long sleep (≥10 h) showed no significant association with CVD risk in either health group. 

**Figure 1:**
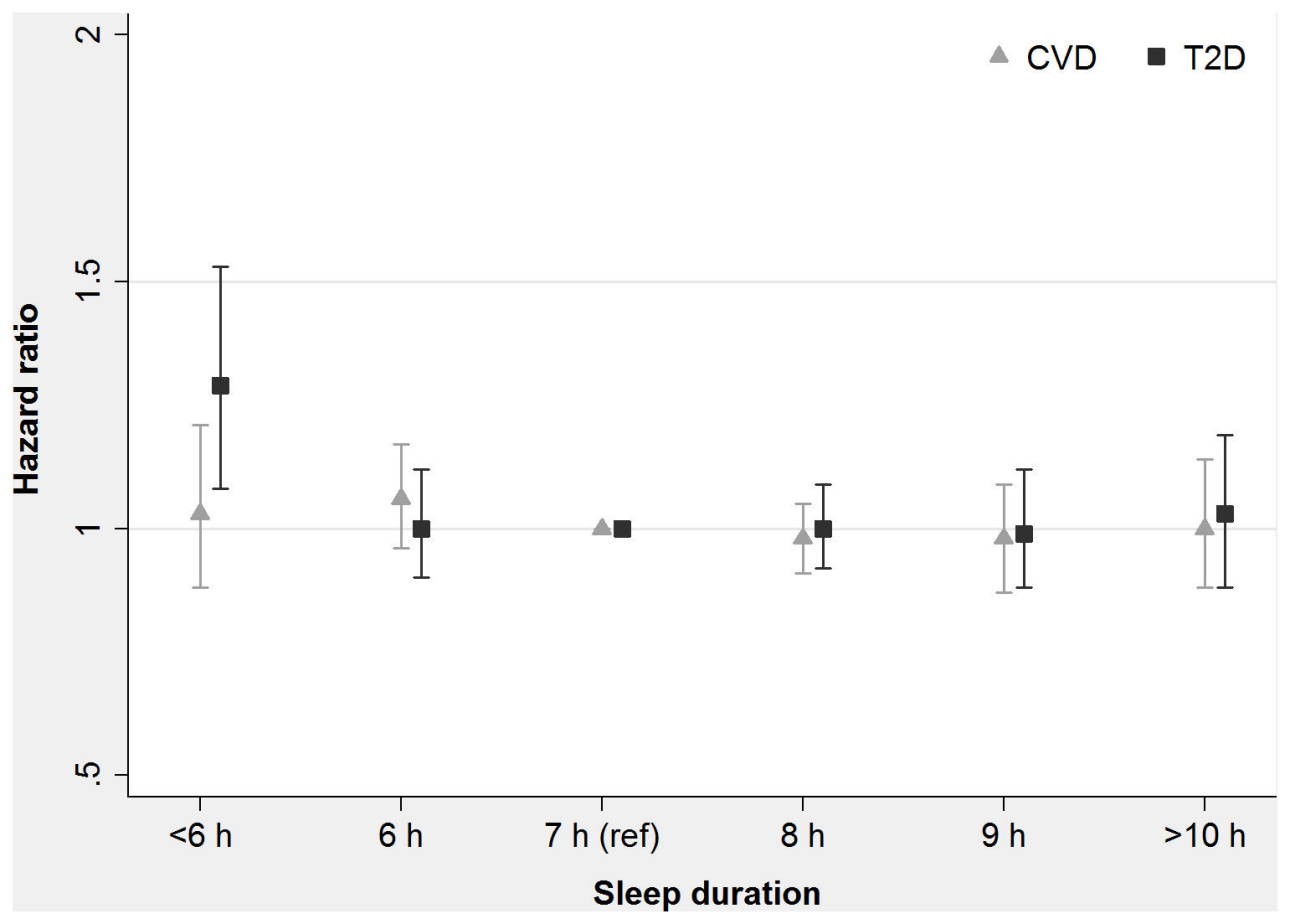
Hazard ratio for incident cardiovascular disease (CVD) and type 2 diabetes by sleep duration category in the 45 and Up Study. The plots shows hazard ratios with 95% confidence intervals for the specified category of sleep duration, compared with 7 h, after adjusting for potential confounders. Analyses excluded individuals reporting any serious illness at baseline (cancer, heart disease, stroke or diabetes).

Model 3 excluded a further 24,642 individuals reporting either baseline type 2 diabetes or current/past cancer. Among the remaining 156,902 individuals, there were 4,852 incident cases of CVD (4,214 cases of ischaemic heart disease, 587 cases of ischaemic stroke and 51 cases of peripheral vascular disease). In this model, which excluded individuals with a range of serious illnesses at baseline, no sleep duration category was significantly associated with incident CVD (all *P*>0·2; see Table 2 and Figure 1) and there was also no evidence of interaction between sleep duration and health status upon CVD risk (*P*_interaction_=0·1).

**Table 2:** Association of sleep duration with incident cardiovascular disease (CVD) in the 45 and Up Study.

	**Model 1^*a*^**	**Model 2^*b*^**		**Model 3^*c*^**	
Sleep duration	HR**^*d*^**	*Healthy* HR	*Less Healthy* HR	HR	Events/py**^*e*^**
<6 h	1·16 (1·01-1·34)*	0·92 (0·75-1·14)	1·38 (1·12-1·70)**	1·03 (0·88-1·21)	165/11,332
6 h	1·09 (1·00-1·19)	1·06 (0·96-1·18)	1·10 (0·93-1·31)	1·06 (0·96-1·17)	629/44,275
7 h	1	1	1	1	1,228/93,789
8 h	1·01 (0·94-1·07)	0·94 (0·87-1·02)	1·18 (1·03-1·35)*	0·98 (0·91-1·05)	1,956/150,222
9 h	1·01 (0·92-1·11)	0·95 (0·85-1·06)	1·14 (0·97-1·34)	0·98 (0·89-1·09)	555/40,405
≥10 h	1·10 (0·98-1·22)	0·97 (0·83-1·14)	1·17 (1·00-1·39)	1·00 (0·88-1·14)	319/20,647

Notes: Individuals with missing data for sleep duration, age, sex, physical functioning or baseline illness were excluded from all analyses. * *P*<0·05. ** *P*<0·01.

***^a^*** Model 1 (N=181,544) included all eligible participants except those reporting CVD at baseline, and was adjusted for age and sex. ***^b^*** Model 2 (N=181,544) included all eligible participants except those reporting CVD at baseline, and was adjusted for age, sex, education, marital status, residential remoteness, alcohol consumption, smoking status, health insurance status, income, body mass index, physical activity and baseline health status. Results are presented separately for healthy and less healthy participants, as significant interaction between sleep duration and health status was observed. ***^c^*** Model 3 (N=156,902) excluded all participants reporting any serious illness at baseline (current/past cancer, or diagnosed/ treated heart disease, stroke or type 2 diabetes), and was adjusted for the same set of covariates as Model 2. ***^d^*** Hazard ratio with 95% confidence interval, representing the estimated risk of CVD for the specified category of sleep duration, compared with the reference category of 7 h. ***^e^*** Number of cardiovascular disease (coronary heart disease, ischaemic stroke or peripheral vascular disease) hospitalisations/total person years at risk during follow-up.

Analyses of type 2 diabetes using Model 1 and 2 included 192,728 individuals without type 2 diabetes, among whom there were 4,648 recorded incident cases of type 2 diabetes. Model 1, which was adjusted only for age and sex, showed association of short sleep (<6 h: HR=1·28 [95% CI: 1·10-1·49], *P*=0·002) with the development of type 2 diabetes. Model 2, which was adjusted for a range of additional covariates, showed similarly positive association of short sleep with type 2 diabetes (HR=1·23 [95% CI: 1·06-1·44], *P*=0·007), with no interaction of sleep duration with baseline health status (*P*_interaction_=0·8). Model 3 excluded a further 35,826 individuals reporting CVD or current/past cancer. Among the remaining 156,902 individuals, there were 3,641 recorded incident cases of type 2 diabetes. In this model, similar association of short sleep with elevated type 2 diabetes was observed (HR=1·29 [95% CI: 1·08-1·53], *P*=0·004), with no confounding or effect modification by baseline health status (*P*_interaction_=0·2) (Table 3).

**Table 3:** Association of sleep duration with incident type 2 diabetes in the 45 and Up Study.

	**Model 1^*a*^**	**Model 2^*b*^**	**Model 3^*c*^**	
Sleep duration	HR**^*d*^**	HR	HR	Events/py**^*e*^**
<6 h	1·28 (1·10-1·49)**	1·23 (1·06-1·44)**	1·29 (1·08-1·53)**	147/11,335
6 h	1·03 (0·93-1·13)	1·01 (0·91-1·12)	1·00 (0·90-1·12)	438/44,521
7 h	1	1	1	921/94,111
8 h	1·01 (0·93-1·08)	1·00 (0·93-1·08)	1·00 (0·92-1·09)	1,500/150,728
9 h	1·00 (0·91-1·11)	1·00 (0·90-1·11)	0·99 (0·88-1·12)	410/40,565
≥10 h	1·09 (0·97-1·23)	1·05 (0·93-1·19)	1·03 (0·88-1·19)	225/20,737

Notes: ** *P*<0·01.

***^a^*** Model 1 (N=192,728) included all eligible participants except those reporting type 2 diabetes at baseline, and was adjusted for age and sex. ***^b^*** Model 2 (N=192,728) included all eligible participants except those reporting type 2 diabetes at baseline, and was adjusted for age, sex, education, marital status, residential remoteness, alcohol consumption, smoking status, health insurance status, income, body mass index, physical activity and baseline health status. ***^c^*** Model 3 (N=156,902) excluded all participants reporting any serious illness at baseline (current/past cancer, or diagnosed/ treated heart disease, stroke or type 2 diabetes), and was adjusted for the same set of covariates as Model 2. ***^d^*** Hazard ratio with 95% confidence interval, representing the estimated risk of type 2 diabetes for the specified category of sleep duration, compared with the reference category of 7 h. ***^e^*** Number of incident type 2 diabetes cases recorded/total person years at risk during follow-up.

### Meta-analysis of prospective studies assessing the relationship between short sleep and incident diabetes

**Figure 2:**
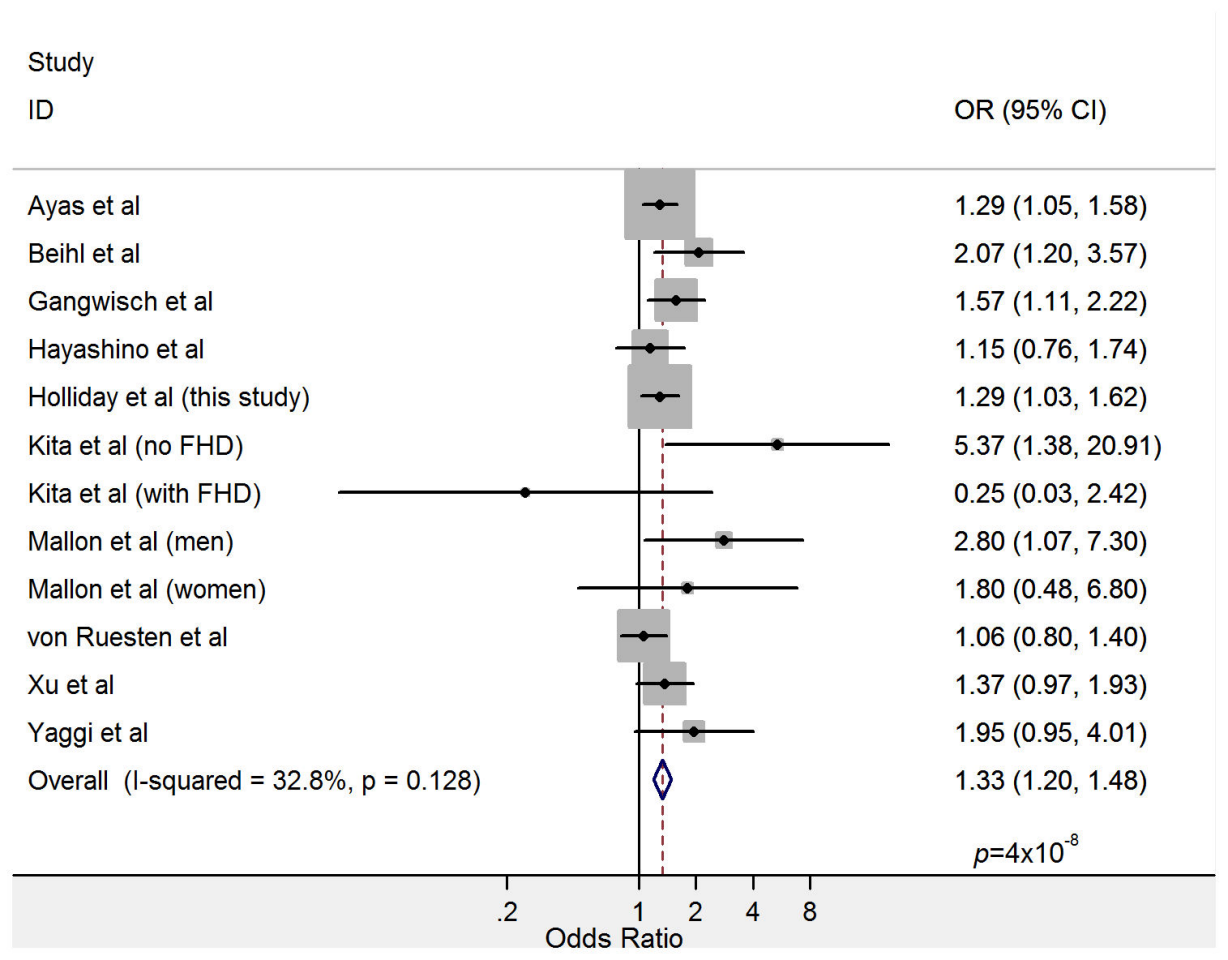
Forest plot showing the relationship between short sleep and incident diabetes in a meta-analysis of prospective studies including 447,124 total participants. Point estimates and 95% confidence intervals are shown as black circles and solid lines, respectively. Grey rectangles indicate the relative weight assigned to individual studies reflecting sample size. Heterogeneity metrics and the summary estimate are shown in the final rows.

Results from ten studies qualified for inclusion in the meta-analysis of association between short sleep and diabetes (see Table 4). These included 447,124 participants of predominantly European ancestry, excepting two studies (combined *N*=10,079) conducted in Japanese populations [28]. One study reported results separately for men and women [18], and another for participants with and without a family history of diabetes [29]; for these, subgroup-specific estimates were included as separate studies. All studies defined the outcome as either incident type 2 diabetes or incident total diabetes, of which ~90-95% is expected to represent type 2 diabetes [30]. Effect estimates from individual studies and the meta-analysis are shown in Figure 2. There was low between-study heterogeneity (*I* ^2^=32·8%: *p*=0·13), and the overall association of short sleep with diabetes risk was highly significant (OR=1·33 [95%CI: 1·20, 1·48); *p*=4x10^-8^), with no evidence of publication bias (Egger’s test *p*=0·15).

**Table 4:** Details of prospective studies included in the meta-analysis assessing the relationship of short sleep with incident diabetes.

**Study**	**Country**	**Sample size**	**Sex**	**Follow-up Duration**	**Exposure** **(short sleep /referent)**	**Outcome**	Covariate adjustments^*^
Ayas et al [14]	USA	70,026	Women	10 y	≤ 5 h vs 8 h	Total diabetes	1, 2, 9, 14, 18, 19, 21, 28, 34, 36, 37
Beihl et al [17]	USA	654	Both	5 y	≤ 7 h vs 8 h	Type 2 diabetes	1, 4, 8, 10, 14, 21, 33, 36
Gangwisch et al [16]	USA	8,992	Both	8-10 y	≤ 5 h vs 7 h	Total diabetes	1, 2, 9, 10, 13, 26, 28
Hayashino et al [28]	Japan	6,509	Both	Median 4·2 y	< 6 h vs 6-7 h	Total diabetes	1, 18, 21, 28, 29, 33, 36, 38
Holliday et al (this study)	Australia	180,891	Both	Mean 2·3 y	< 6 h vs 7-8 h	Type 2 diabetes	1, 2, 5, 10, 16, 22, 26, 28, 31, 33, 36
Kita et al [29]	Japan	3,570	Both	3-5 y	≤ 5 h vs > 7 h	Total diabetes	1, 2, 6, 10, 15, 23, 28, 30, 33, 34, 36, 41. *Notes*: effects reported separately for those with and without a family history of diabetes.
Mallon et al [18]	Sweden	1,170	Both	12 y	≤ 5 h vs > 5 h	Total diabetes	1, 2, 9, 21, 25, 27, 36, 37. *Notes*: effects reported separately for men and women.
Von Ruesten et al [39]	Germany	23,620	Both	9-13 y	< 6 h vs 7-8 h	Type 2 diabetes	2, 3, 6, 7, 10, 11, 17, 20, 21, 24, 28, 33, 35, 36, 40
Xu et al [15]	USA	174,542	Both	7-10 y	< 5 h vs 7-8 h	Total diabetes	1, 2, 5, 7, 10, 12, 13, 14, 26, 33, 36. *Notes*: Diabetes cases diagnosed within the first 3-4 y of follow-up were excluded. Estimate is from an analysis of non-smoking, nonobese, physically active, healthy individuals.
Yaggi et al [40]	USA	1,139	Men	15-17 y	≤ 5 h vs 7 h	Total diabetes	1, 10, 21, 32, 36, 39

* *Covariate definitions*: 1: age, 2: alcohol use, 3: antidepressant intake, 4: baseline glucose tolerance, 5: baseline health status, 6: BMI, 7: caffeine use, 8: clinic, 9: depression, 10: education, 11: employment status, 12: energy intake, 13: ethnicity, 14: family history of diabetes, 15: fasting plasma glucose, 16: health insurance, 17: health satisfaction, 18: high cholesterol, 19: hormone replacement, 20: hyperlipidaemia, 21: hypertension, 22: income, 23: job stress, 24: life satisfaction, 25: living arrangement, 26: marital status, 27: obesity, 28: physical activity, 29: potential diabetes history, 30: rate of sedentary work, 31: residential remoteness, 32: self-rated health, 33: sex, 34: shift work, 35: sleeping disorders, 36: smoking, 37: snoring, 38: study intervention, 39: waist circumference, 40: waist-to-hip ratio, 41: working hours per week.

## Discussion

To our knowledge, this represents the largest longitudinal study assessing relationships between sleep duration and future cardiometabolic disorders. After accounting for baseline illness or functional impairment, there was no significant association between baseline sleep duration and future cardiovascular disease. In contrast, short sleep duration (<6 h) was prospectively related to future, incident type 2 diabetes; this result was corroborated via meta-analysis of 10 prospective studies including more than 440,000 total participants.

### Relationship of sleep duration with future cardiovascular disease

This study offers insights into previously reported relationships between sleep duration and cardiovascular disease. The presence of CVD is known to affect an individual’s sleeping pattern [31], and CVD often co-exists with other health conditions influencing sleep duration and quality, such as obesity, hypertension and type 2 diabetes [32]. However, few previous prospective studies reporting associations between sleep duration and cardiovascular outcomes have incorporated comprehensive measures of baseline health or functional capacity. Some used binary variables reflecting the presence or absence of a particular health condition (e.g. hypertension, dyslipidaemia, cardiac disease, diabetes). But among those with a specific illness, there will be substantial variation in the severity of disease, level of sleep disturbance and underlying cardiovascular disease risk. For this reason, adjustment simply for the presence or absence of disease is unlikely to deal adequately with confounding. 

Obviously, the assessment of the nature of any exposure-outcome relationship is not dependent solely on any one piece of evidence, but on a broader consideration of clinical, biological, epidemiological and other data. The general lack of consistency of findings regarding sleep duration and cardiovascular disease, the known relationships of sleep disturbance to baseline health [33], and lack of association of sleep duration with CVD risk in healthy individuals in our study, suggests that observed relationships between short and long sleep and CVD risk may largely reflect the effects of pre-existing disease. 

### Relationship of sleep duration with future type 2 diabetes

In contrast, our detection of a significant association between short sleep duration (<6 h) and the development of type 2 diabetes was robust to all exclusions and covariate adjustments. This association was not only statistically significant, but also appears to be clinically important, with short sleep associated with an estimated 30% increased risk of T2D. Thus, our result reflects more than the statistical association of a clinically trivial effect due to large sample size. The positive association is also compelling given that the same exclusions and covariate adjustments abolished the association between short sleep and CVD. Comprehensive modelling provided no evidence for modification or confounding of the type 2 diabetes association by baseline health status. Furthermore, we found no evidence for an association of long sleep with type 2 diabetes, which is widely suggested to reflect residual confounding. These points all suggest a lack of residual confounding, supporting a causal relationship between short sleep and type 2 diabetes risk. While we cannot completely exclude the possibility of residual confounding in the studies retrieved for our meta-analysis, the high consistency between the results of our study and those of previous studies suggests an absence of systematic confounding in the retrieved studies. The low heterogeneity and lack of publication bias supports the validity of the meta-analysis result. 

Well-controlled laboratory studies also provide credible evidence that sleep loss impairs glucose clearance and insulin sensitivity, increasing type 2 diabetes risk. Sleep deprivation has been shown to reduce glucose tolerance and insulin sensitivity [34]. Lack of sleep also elevates concentrations of the appetite-stimulating hormone ghrelin and decreases levels of the satiety hormone leptin [35,36], leading to increased hunger and appetite [37]. Sleep restriction is also associated with disrupted cortisol and growth hormone secretion, predisposing to insulin resistance and hepatic glucose output [38], both of which increase type 2 diabetes risk. 

Strengths of this study include the prospective design, comprehensive data linkage, a large study population containing thousands of objectively measured events, and an internal control (analyses of incident CVD) to demonstrate the effects of residual confounding. The use of such an internal control is unique among observational studies to date. Limitations of the study include modest response rate and a relatively short period of follow-up, which may have decreased the magnitude of observed associations with either CVD or type 2 diabetes. There is also some degree of measurement error including:

• Exposure misclassification since we were also not able to distinguish between short sleep as a result of restricted bedtime, or sleep disruption due to a sleep disorder; sleep duration was also self-reported.
• For type 2 diabetes, measurement of time to onset was imprecise because diabetes may have developed prior to the hospital admission at which it was ascertained.
• Inability to ascertain individuals who developed type 2 diabetes, but were not admitted to hospital during follow-up.

The first of these (explosure misclassification) is likely to be non-differential with regard to the outcome, and thus will reduce power to detect an association between the exposure and outcome. The second two errors are anticipated to each bias estimated effects towards the null. Those who developed diabetes prior to the hospitalisation at which diabetes was ascertained will have an erroneously *longer* time to onset coded in the Cox proportional hazards model, thus reducing the apparent hazard (per unit time) associated with the exposure. Further, inability to ascertain diabetes in cases not hospitalised during follow-up means these unascertained diabetic cases were included in the reference group for the entire follow-up period. This will also reduce the apparent hazard associated with the exposure. Taken together, each of these errors is predicted to *reduce* our ability to detect an association between sleep duration and diabetes. This makes the observed association seem all the more robust. Our use of a parallel design and identical cohort for assessing the prospective association of sleep duration with both CVD and type 2 diabetes is unique and provides strong internal support for a causal relationship between short sleep and type 2 diabetes. These results have relevance for public health programs seeking to reduce chronic disease risk.

## Supporting Information

Figure S1 **Flow diagram detailing the exclusions, sample size and covariates used in each analysis model.**
(DOCX)

Checklist S1 PRISMA checklist for meta-analysis.
(DOC)
