# Supplementary material for: Short Sleep Duration Is Associated with Risk of Future Diabetes but Not Cardiovascular Disease: a Prospective Study and Meta-Analysis
Source: PLoS One. 2013 Nov 25;8(11):e82305. doi: 10.1371/journal.pone.0082305 (PMC3840027; doi:10.1371/journal.pone.0082305)
Supplement: Figure S1 — Flow diagram detailing the exclusions, sample size and covariates used in each analysis model. (DOCX) [file pone.0082305.s001.docx]

**Flow Diagram for Analyses of Cardiovascular Disease and Type 2 Diabetes in the 45 and Up Study**

## Type 2 Diabetes (T2D)

Available in baseline dataset (n=241,949)

Excluded

♦ Missing data for sleep duration, age, sex, physical functioning or serious illness (n=29,561)

♦ Prevalent T2D at baseline (n=19,660)

Eligible for **T2D** analysis

(n=192,728; with 4,648 T2D events)

## Cardiovascular Disease (CVD)

Excluded

♦ Missing data for sleep duration, age, sex, physical functioning or serious illness (n=29,561)

♦ Prevalent CVD at baseline (n=30,844)

Eligible for **CVD** analysis

(n=181,544; with 5,814 CVD events)

Analysed (n=156,902; 4,852 CVD events)
♦ Adjusted for the same covariates as Model 2.

♦ Adjusted for age, sex, education, marital status, residential remoteness, alcohol, smoking, health insurance, income, BMI, physical activity, baseline health.

♦ Adjusted for age and sex

## CVD Model 1

## CVD Model 2

Excluded

♦ Prevalent diabetes or current/past cancer at baseline (n=24,642)

## CVD Model 3

Analysed (n=156,902; 3,641 T2D events)
♦ Adjusted for the same covariates as Model 2.

♦ Adjusted for age, sex, education, marital status, residential remoteness, alcohol, smoking, health insurance, income, BMI, physical activity, baseline health.

♦ Adjusted for age and sex

## T2D Model 1

## T2D Model 2

Excluded

♦ Prevalent CVD or current/past cancer at baseline (n=35,826)

## T2D Model 3
